# Criminal Spread of Infection

**Published:** 1883-03

**Authors:** 


THE CRIMINAL SPREAD OF INFECTION.—Judge Dixon, of New Jersey, in a recent charge to the grand jury of Paterson, called their attention to the case of a man employed at the pest house as nurse to a small-pox patient, and who, having the germs of the infectious disease about him, went recklessly to his family, communicating the disease to his children, one of whom died. In commenting on this case, he said: "If a man, conscious that he carries about with him the germs or a contagious disease, recklessly exposes the health and lives of others, he is a public nuisance and a criminal, and may be held answerable for the results of his conduct. If death occurs through his recklessness he may be indicted for manslaughter. It is held that where a person knowingly communicates a contagious disease to another, and death results, the crime is that of manslaughter." Judge Dixon furthermore added: "The man may be indicted also for spreading the disease by conscious exposure of others thereto by his presence in public places, such as on the street, in halls, etc. He might be indicted as a public nuisance for endangering the public health in this way, even if no consequences had followed. The law provides some penalty for such offenses against the public safety."—*Boston Med. and Surg. Jour.*

